# Cannabidiol and Sports Performance: a Narrative Review of Relevant Evidence and Recommendations for Future Research

**DOI:** 10.1186/s40798-020-00251-0

**Published:** 2020-07-06

**Authors:** Danielle McCartney, Melissa J. Benson, Ben Desbrow, Christopher Irwin, Anastasia Suraev, Iain S. McGregor

**Affiliations:** 1 The University of Sydney, Faculty of Science, School of Psychology, Sydney, New South Wales 2050 Australia; 2 The University of Sydney, Lambert Initiative for Cannabinoid Therapeutics, Sydney, New South Wales Australia; 3 The University of Sydney, Brain and Mind Centre, Sydney, New South Wales Australia; 4 School of Allied Health Sciences, Griffith University, Gold Coast, Queensland Australia; 5 Menzies Health Institute Queensland, Gold Coast, Queensland Australia

**Keywords:** Cannabidiol, CBD, Cannabis, Cannabinoid, Athletic performance, Exercise

## Abstract

Cannabidiol (CBD) is a non-intoxicating cannabinoid derived from *Cannabis sativa*. CBD initially drew scientific interest due to its anticonvulsant properties but increasing evidence of other therapeutic effects has attracted the attention of additional clinical and non-clinical populations, including athletes. Unlike the intoxicating cannabinoid, Δ^9^-tetrahydrocannabinol (Δ^9^-THC), CBD is no longer prohibited by the World Anti-Doping Agency and appears to be safe and well-tolerated in humans. It has also become readily available in many countries with the introduction of over-the-counter “nutraceutical” products. The aim of this narrative review was to explore various physiological and psychological effects of CBD that may be relevant to the sport and/or exercise context and to identify key areas for future research. As direct studies of CBD and sports performance are is currently lacking, evidence for this narrative review was sourced from preclinical studies and a limited number of clinical trials in non-athlete populations. Preclinical studies have observed robust anti-inflammatory, neuroprotective and analgesic effects of CBD in animal models. Preliminary preclinical evidence also suggests that CBD may protect against gastrointestinal damage associated with inflammation and promote healing of traumatic skeletal injuries. However, further research is required to confirm these observations. Early stage clinical studies suggest that CBD may be anxiolytic in “stress-inducing” situations and in individuals with anxiety disorders. While some case reports indicate that CBD improves sleep, robust evidence is currently lacking. Cognitive function and thermoregulation appear to be unaffected by CBD while effects on food intake, metabolic function, cardiovascular function, and infection require further study. CBD may exert a number of physiological, biochemical, and psychological effects with the potential to benefit athletes. However, well controlled, studies in athlete populations are required before definitive conclusions can be reached regarding the utility of CBD in supporting athletic performance.

## Key Points

- CBD has been reported to exert a number of physiological, biochemical, and psychological effects that have the potential to benefit athletes.
- The available evidence is preliminary, at times inconsistent, and largely based on preclinical studies involving laboratory animals.
- Rigorous, controlled investigations clarifying the utility of CBD in the sporting context are warranted.

## Introduction

*Cannabis sativa* contains numerous chemical compounds with potential bioactive effects, including at least 144 cannabinoids [56, 76]. The most studied of the cannabinoids are Δ^9^-tetrahydrocannabinol (Δ^9^-THC), renowned for its distinctive intoxicating effects [73, 123], and cannabidiol (CBD)—a non-intoxicating cannabinoid that is particularly enriched in industrial hemp cultivars grown for seed and fibre [61]. CBD was first isolated in 1940 and initially considered to be biologically inactive, with no apparent therapeutic or “subjective” drug effects [1]. However, in 1973, Carlini et al. [27] demonstrated anticonvulsant effects of CBD in a preclinical model, which were later mirrored in humans suffering from intractable epilepsy [46]. A subsequent rise in research into CBD [206] has uncovered interactions with numerous molecular targets [92] and a range of potential therapeutic applications [138]. Following successful phase 3 clinical trials [53, 54, 172], the oral CBD solution, Epidiolex®, has also recently gained Food and Drug Administration approval as a regulated prescription medication to treat certain forms of paediatric epilepsy.

Recently, interest in CBD has intensified among the general population as evidenced by an exponential rise in internet searches for ‘CBD’ in the United States (USA) [108]. Some professional athletes (e.g. golfers, rugby players) also appear to be using CBD (e.g. ‘Team cbdMD’ https://www.cbdmd.com/), despite there being no published studies demonstrating beneficial effects on sport or exercise performance. In many jurisdictions, including the USA and Europe, access to regulated, prescription CBD (i.e. Epidiolex®) is limited to patients with intractable epilepsy. However, a wide range of low dose (e.g. 5–50 mg·d^−1^) CBD-containing “nutraceuticals” (primarily in oil or capsule form) have become readily available online and over-the-counter (e.g. pharmacies, health food stores) [20, 125]. This includes some varieties that are marketed specifically to recreational and elite athletes (e.g. cbdMD, fourfivecbd). The use of these products is likely to become even more widespread if the World Health Organization’s recommendation that CBD no longer be scheduled in the international drug control conventions is adopted by the United Nations member states [201].

Cannabis has been prohibited in all sports during competition since the World Anti-Doping Agency first assumed the responsibility of establishing and maintaining the list of prohibited substances in sport 15 years ago [89]. In 2018, however, CBD was removed from the Prohibited List [199], presumably on the basis of mounting scientific evidence that the cannabinoid is safe and well-tolerated in humans [16, 169], even at very high doses (e.g. 1500 mg·day^−1^ or as an acute dose of 6000 mg) [170]. While several recent reviews have described the impact of cannabis on athlete health and performance [99, 176, 188], the influence of CBD alone has yet to be addressed.

The aim of this narrative review was to explore evidence on the physiological, biochemical, and psychological effects of CBD that may be relevant to sport and/or exercise performance and to identify relevant areas for future research. Given the absence of studies directly investigating CBD and sports performance, this review draws primarily on preclinical studies involving laboratory animals and a limited number of clinical trials involving non-athlete populations.

## Cannabidiol (CBD): Molecular Targets, Pharmacokinetics and Dosing

### Molecular Targets

The distinctive intoxicating effects of Δ^9^-THC (as well as some of its therapeutic effects) involve the activation of CB_1_R (the cannabinoid type 1 receptor) [12]. This ubiquitous receptor is expressed throughout the central nervous system, the peripheral nervous system, and in the cardiovascular system, gastrointestinal (GI) tract, skeletal musculature, liver, and reproductive organs [205]. Unlike Δ^9^-THC, CBD is not an agonist of CB_1_R, although it may act as a negative allosteric modulator (NAM) at this site (i.e. decreasing the potency and/or efficacy of other ligands without activating the receptor itself) [92, 106]. Δ^9^-THC also acts as an agonist at CB_2_R (the cannabinoid type 2 receptor) [12] and there is emerging evidence of CBD functioning as a partial agonist at this site [171]. CB_2_R is primarily located on immune system cells but is also expressed in the cardiovascular system, GI tract, bone, liver, adipose tissue, and reproductive organs [205]. CBD may also influence the endocannabinoid system indirectly via the inhibition of fatty acid amide hydrolase (FAAH), a key enzyme involved in the degradation of the principle endocannabinoid signalling molecule, anandamide (AEA) [92, 110]. The inhibition of FAAH is predicted to lead to an increase in brain and plasma concentrations of AEA, which acts as a partial agonist at CB_1_R and CB_2_R, thereby increasing endocannabinoid tone [92, 110]. Increases in endocannabinoid tone may also occur as a result of CBD inhibiting AEA transport via effects on fatty acid-binding proteins (and this mechanism may have more relevance than FAAH inhibition in humans) [57].

CBD also interacts with many other non-endocannabinoid signalling systems [92]. Briefly, at concentrations ≤ 10 μM, CBD has been reported to interact with the serotonin 1A [5-HT_1A_] receptor, the orphan G protein-coupled receptor 55, as well as the glycine, opioid, and peroxisome proliferator-activated receptors, various ion channels (e.g. the transient potential vanilloid receptor type 1 channel [TRPV1] and other transient potential vanilloid channels) and various enzymes (e.g. cyclooxygenase (COX)1 and COX2, cytochrome P450 enzymes) [11, 92] (see Ibeas et al. [92] for review). CBD also possesses antioxidant properties [92].

It is important to recognise that the molecular targets of CBD are still being established, with many of those identified in in vitro cellular assays still to be validated as occurring in vivo. As such, the functional relevance of many of these interactions remains to be established.

### Pharmacokinetics

CBD is often consumed orally as oil; however, it can also be ingested in other forms (e.g. gel capsules, tinctures, beverages, and confectionery products) and applied topically [20, 125]. High concentration CBD “vape oils” (i.e. for use in e-cigarette devices) are also available in some countries, as are some CBD-dominant forms of cannabis (sometimes known as “light cannabis”) that can be smoked or vaporised [20, 125]. Pure, synthetic, crystalline CBD was also vaporised in a recent laboratory study [160].

Taylor et al. [170] recently conducted a comprehensive analysis of oral CBD oil pharmacokinetics in healthy participants. When administered as a single, oral dose (1500–6000 mg), the time to reach peak plasma concentrations (*t*_max_) was ~4–5 h and the terminal half-life was ~14–17 h. Although *t*_max_ did not increase dose-dependently in this investigation [170], another study [19], involving a much lower oral dose of CBD (300 mg), did indicate a shorter *t*_max_ (i.e. ~2–3 h). Peak plasma concentrations (*C*_max_) were ~0.9–2.5 μM in Taylor et al. [170], but increased ~4.9-fold when CBD was administered with a high-fat meal (i.e. ~5.3 μM at 1500 mg dose) [170]. Both studies observed a large amount of inter-individual variation in pharmacokinetic responses [19, 170].

The pharmacokinetics of inhaled CBD are yet to be well characterised. However, smoked “light cannabis” (with a lower Δ^9^-THC and higher CBD content than other varieties) has been reported to elicit high serum CBD concentrations at 30 min post-treatment (that decline over time) [146]. A recent study in which participants vaporised 100 mg of CBD likewise observed high blood CBD concentrations 30 min post-treatment [160]. As neither study collected blood samples within < 30 min of CBD administration, *t*_max_ and *C*_max_ are unknown [146, 160].

CBD is metabolised by several cytochrome P [CYP] 450 enzymes (e.g. CYP3A4, CYP2C9, CYP2C19) which convert it to a number of primary and secondary metabolites (e.g. 7-OH-CBD, 6-OH-CBD, and 7-COOH-CBD) [177]. Complex pharmacokinetic interactions may occur when CBD is co-administered with other drugs (e.g. Δ^9^-THC) and dietary constituents (e.g. caffeine) that also utilise these enzymes [6, 163].

### Interspecies Dose Conversions

Given the number of preclinical studies involving animal models that will be discussed in this review, it is important to consider interspecies dose equivalence (Table 1). The USA Food and Drug Administration [30] recommend the following approach to interspecies dose conversion:

$$ \mathrm{HED}\left(\mathrm{mg}\cdot {\mathrm{kg}}^{\hbox{-} 1}\right)={\mathrm{Dose}}_{\mathrm{Animal}}\left(\mathrm{mg}\cdot {\mathrm{kg}}^{\hbox{-} 1}\right)\times \left(\frac{{\mathrm{Km}}_{\mathrm{Animal}}}{{\mathrm{Km}}_{\mathrm{Human}}}\right) $$

Where HED is the human equivalent dose and Km is a correction factor estimated by dividing the average body mass (BM) of the species (60, 0.020 and 0.150 kg for 11 humans, mice and rats respectively) and by its surface area (see: Nair, et al. [134] for 12 further details).

Differences between systemic and oral dosing should also be considered [9]. Intraperitoneal (i.p.) dosing is often used in animal studies and has been reported to elicit *C*_max_ values ~7-fold higher than oral dosing in mice [52]. Thus, an “oral equivalent dose” can be approximated by multiplying the i.p. dose by seven [9] (Table 1). Intravenous (i.v.) dosing will produce even higher plasma CBD concentrations; however, the authors are not aware of any published data that would facilitate conversion between i.v. and oral dosing in rodents. Please note that these values are intended as a guide only and subject to limitations (e.g. interspecies differences in drug potency and receptor expression/configuration).

**Table 1:** Oral human equivalent CBD doses from mouse and rat intraperitoneal doses

Mouse to Human CBD Dose Conversion		Rat to Human CBD Dose Conversion	
Mouse Dose (mg·kg^-1^, i.p.)	HED (mg, p.o.)	Rat Dose (mg·kg^-1^, i.p.)	HED (mg, p.o.)
1	34	1	68
5	170	5	340
10	341	10	681
20	681	20	1362
30	1021	30	2043
60	2043	60	4086

Each HED is based on a body mass of 60 kg and calculated as per the methods described in 2.3 *Dose Conversions*. The highest documented acute oral CBD dose in humans is 6000 mg; the highest documented chronic oral CBD dose in humans is 1500 mg [169]. HED: Human Equivalent Dose; i.p.: Intraperitoneal; p.o.: Oral

## Cannabidiol (CBD) in Sport and Exercise Performance

### Literature Search Methodology

The clinical and preclinical literature was reviewed to identify studies investigating the effects of CBD that might be relevant within a sport and/or exercise context. The online databases PubMed (MEDLINE), Web of Science (via Thomas Reuters), and Scopus were searched between April and October of 2019 using terms such as: ‘cannabinoid’ ‘cannabidiol’, ‘CBD’ and ‘cannabis’. This review focuses primarily on effects that have been demonstrated in vivo and generally avoids attempting to predict functional effects on the basis of target-oriented in vitro data, given the numerous molecular targets of CBD [92] and the fact that exercise itself induces complex biochemical changes. Nonetheless, some potential interactions are noted. As our intent was to summarise evidence on a range of potentially relevant topics, rather than provide a detailed assessment of the literature, the reader will be directed to more focused reviews, where appropriate. All doses described are oral and acute (single), unless otherwise stated.

### Exercise-Induced Muscle Damage—Muscle Function, Soreness, and Injury

Exercise, particularly when strenuous, unfamiliar, and/or involving an eccentric component, can cause ultrastructural damage to skeletal muscle myofibrils and the surrounding extracellular matrix [36, 59]. This exercise-induced muscle damage (EIMD) impairs muscle function and initiates an inflammatory response [59]. While inflammation is integral to EIMD repair, regeneration, and adaptation [59], excessive inflammation may contribute to prolonged muscle soreness and delayed functional recovery [7, 158].

CBD modulates inflammatory processes [21]. In preclinical models of acute inflammation, CBD has been reported to attenuate immune cell accumulation (e.g. neutrophils, lymphocytes macrophages) [102, 130, 149, 186], stimulate production of anti-inflammatory cytokines (e.g. interleukin (IL)-4, IL-10) [190, 191, 23] and inhibit production of pro-inflammatory cytokines (e.g. IL-1β, IL-6, IL-8, tumour necrosis factor (TNF)-α) [10, 50, 55, 62, 63, 113, 130, 149, 154, 186] and reactive oxygen species [62, 130, 186]. Models demonstrating such effects have included lung injury induced by chemical treatment [149] and hypoxic–ischemia (HI) [10]; liver injury induced by ischemia-reperfusion [63, 130] and alcohol feeding [186]; myocardial [55] and renal [62] ischemia-reperfusion injuries; surgically induced oral lesions [102]; chemically induced osteoarthritis [145]; spinal cord contusion injury [113], and colitis [23, 50, 154] (see Burstein [24] for review). Anti-inflammatory effects are generally observed at higher CBD doses in vivo (e.g. ≥ 10 mg·kg^−1^, i.p.); although, lower doses (e.g. ~1.5 mg·kg^−1^, i.p.) have indicated efficacy in some studies [145]. Research investigating the effects of CBD on inflammation in humans is limited and inconclusive [94, 133].

In terms of muscle-specific inflammation, one preclinical study has investigated the effect of high-dose CBD (i.e. 60 mg·kg^−1^·d^−1^, i.p.) on transcription and synthesis of pro-inflammatory markers (i.e. IL-6 receptors, TNF-α, TNF-β1, and inducible nitric oxide synthase) in the gastrocnemius and diaphragm of dystrophic MDX mice (a mouse model of Duchenne muscular dystrophy) [91]. In this investigation, CBD attenuated mRNA expression of each marker and reduced plasma concentrations of IL-6 and TNFα. Improvements in muscle strength and coordination, as well as reductions in tissue degeneration, were also reported at this dose. Lower, but still relatively high, CBD doses (20–40 mg·kg^−1^·day^−1^, i.p.) had no functional benefits [91]. Of course, it is important to recognise that EIMD and muscular dystrophy differ in their pathophysiology, and so the effects observed in MDX mice may involve mechanisms less relevant to EIMD (e.g. skeletal muscle differentiation, autophagy) [91].

While CBD could potentially aid in muscle recovery, other anti-inflammatory agents, such as ibuprofen (a non-steroidal anti-inflammatory drug [NSAID]) have been reported to attenuate exercise-induced skeletal muscle adaptation [120]. The precise mechanism(s) underpinning these effects have not been fully elucidated, although it may be that the prevention of inflammation inhibits angiogenesis and skeletal muscle hypertrophy [120]. Human trials also suggest that ibuprofen may not influence EIMD, inflammation, or soreness [144, 175]. Thus, if CBD exerts its effects via similar mechanisms, it could possibly attenuate the benefits of training without influencing muscle function or soreness. Future studies investigating this are clearly warranted to clarify such issues and elucidate the potential benefits of CBD.

### Neuroprotection—Concussion and Subconcussion

Recent estimates suggest that 6–36% of high school and collegiate athletes in the USA have experienced more than one concussion [72], potentially predisposing them to long-term neurodegenerative diseases [72] and an increased risk of suicide [64]. Concussion is a distinct form of mild traumatic brain injury (TBI) in which a biomechanical force temporarily disrupts normal brain functioning causing neurological–cognitive–behavioural signs and symptoms [97]. Similar injuries that do not produce overt (acute) signs or symptoms are termed “subconcussions” [97]. In TBI, the primary injury occurs as a result of the biomechanical force; secondary injury is then sustained through a complex cascade of events, including HI, cerebral oedema, increased intracranial pressure, and hydrocephalus [203]. These processes are, in turn, related to a number of detrimental neurochemical changes, including glutamate excitotoxicity, perturbation of cellular calcium homeostasis, excessive membrane depolarisation, mitochondrial dysfunction, inflammation, increased free radicals and lipid peroxidation, and apoptosis [203]. While the primary injury may not be treatable, interventions that attenuate secondary sequelae are likely to be of benefit [203].

Only one study [14] has investigated the biochemical and neuropsychological effects of CBD in an animal model of TBI. Here, C57BL/6 mice were given chronic CBD treatment (3 μg·day^−1^, oral) 1–14 and 50–60 days post- (weight drop) brain insult. CBD attenuated the behavioural (e.g. anxious and aggressive behaviour, depressive-like behaviour, impaired social interactions, pain-related behaviours) and some of the cortical biochemical abnormalities were observed. Specifically, CBD tended to normalise extracellular glutamate, d-aspartate, and γ-aminobutyric acid concentrations in the medial prefrontal cortex, suggesting a reduction in excitotoxicity. However, neuronal damage was not measured directly in this study [14].

Other preclinical studies have investigated the impact of CBD on different animal models of acute neuronal injury, in particular, acute cerebral HI [4, 13, 31, 68, 69, 80, 81, 83, 100, 105, 127, 129, 142, 143, 153]. Studies administering a single (acute) dose of CBD shortly post-HI (e.g. ≤1 h) have produced inconsistent results. For instance, while Garberg et al. [68, 69] found no effect of CBD (1 or 50 mg·kg^−1^, i.v.) on HI-induced neuronal damage in piglets, others observed neuroprotection at similar doses (e.g. 1 mg·kg^−1^, i.v [105, 143]., 1 mg·kg^−1^, s.c [127, 142]., and 5 mg·kg^−1^, i.p [31].) in piglets and rats. When given chronically, or repeatedly within a short timeframe proximal to the HI event, however, CBD appears to be neuroprotective. Effective dosing strategies have varied and included initiating treatment several days pre-HI (e.g. 100 or 200 μg·day^−1^, intracerebroventricular 5 days; Wistar rats [100]), shortly pre- and/or post-HI^1^, and up to 3 days post-HI (e.g. 3 mg·kg^−1^·day^−1^, i.p. 12 days; ddY mice [80]). Thus, chronic CBD treatment may be more effective than acute intervention. While “pre-incident” dosing might also be beneficial, it is noted that in practice, this would require humans at risk of TBI to use CBD chronically as a prophylactic.

The precise mechanism(s) underpinning the neuroprotective effects of CBD are not completely understood (see Campos et al. [25] for review), but may involve decreased inflammation, oxidative stress, and excitotoxicity [142, 143] and increased neurogenesis [129]. Preclinical studies have also demonstrated beneficial effects of CBD in other animal models of neurodegeneration (e.g. transgenic model of Alzheimer’s disease [34, 35], brain iron-overload [47, 48]). Collectively, these data suggest that research investigating the utility of CBD in ameliorating the harmful long-term effects of repeated sports concussions is warranted.

### Nociceptive and Neuropathic Pain

Persistent pain is common in athletes [74]. Nociceptive pain, which includes inflammatory pain, typically occurs with tissue damage; whereas neuropathic pain typically results from a lesion or disease in the somatosensory nervous system [74]. Neuropathic pain is common among para-athletes with spinal cord injuries and can also arise with surgery (e.g. to treat an existing injury) or if there is repetitive mechanical and/or inflammatory irritation of peripheral nerves (e.g. as in endurance sports) [74].

Clinical trials investigating the combined effects of Δ^9^-THC and CBD (e.g. Sativex®) on chronic neuropathic pain have yielded promising initial results [87, 114, 151, 156]. However, the therapeutic effects of CBD administered alone have received limited clinical attention. Preclinical (in vivo) studies investigating the effects of CBD on neuropathic and nociceptive pain are summarised in Table 2. Despite some methodological inconsistencies (e.g. the pain model, period of treatment, route of delivery), most preclinical studies appear to have observed a significant analgesic effect of CBD [29, 39–41, 51, 70, 75, 78], albeit somewhat less pronounced than the effects of Δ^9^-THC [29, 78] (e.g. Hedges’ *g* = 0.8 vs. 1.8 [78]) or of gabapentin (e.g. Hedges’ *g* = 2.0 [78]), a commonly used agent for treating neuropathic pain. Capsazepine co-treatment has also been reported to attenuate CBD-induced analgesia, suggesting that the effect may be mediated, at least in part, by the TRPV_1_ channel [40, 41, 51]. This mechanism is noteworthy as studies have implicated the TRPV_1_ in the development of mechanical hyperalgesia induced by muscle inflammation [66, 140].

**Table 2:** Preclinical studies investigating the effect of CBD on neuropathic and nociceptive pain in vivo

Citation	Animal	Model	Treatment(s)	Treatment Effect
**Neuropathic Pain**				
De Gregorio et al., (2019) [51]	Wistar rats	SNI	5 mg·kg^-1^·d^-1^, s.c. 7 d	CBD sig. decreased mechanical allodynia on Tx day 7.
Casey et al., (2017) [29]	C57BL/6 mice	CCI	30 mg·kg^-1^, s.c.	CBD sig. decreased mechanical allodynia 2 h, but not 0.5, 1, 4 or 6 h, post-Tx compared to baseline.
			0.01, 0.1, 1, 10 or 100 mg·kg^-1^, s.c.	CBD dose-dependently decreased mechanical and cold allodynia.
King et al., (2017) [101]	C57BL/6 mice	CT (Paclitaxel)	0.625–20 mg·kg^-1^, i.p. 15 min prior to CT on days 1, 3, 5 and 7	1 and 20 mg·kg^-1^ CBD sig. attenuated the development of mechanical allodynia measured on Tx days 9 and 14, but not 21.
		CT (Oxaliplatin)	1.25–10 mg·kg^-1^, i.p. 15 min prior to CT on days 1, 3, 5 and 7	1.25–10 mg·kg^-1^ CBD sig. attenuated the development of mechanical allodynia measured on Tx days 2, 4, 7 and 10.
		CT (Vincristine)	1.25–10 mg·kg^-1^, i.p. 15 min prior to CT on days 1, 3, 5 and 7	CBD did not attenuate the development of CT-induced mechanical allodynia measured on Tx days 5, 10, 15 and 22.
Harris et al., (2016) [78]	C57BL/6 mice	CT (Cisplatin)	2 mg·kg^-1^, i.p.	CBD sig. decreased tactile allodynia 1 h post-Tx.
			0.5, 1 or 2 mg·kg^-1^, i.p. 30 min prior to CT every second day for 12 d	CBD did not attenuate the development of CT-induced tactile allodynia measured on Tx days 6, 10 and 12.
Ward et al., (2014) [187]	C57BL/6 mice	CT (Paclitaxel)	2.5 or 5 mg·kg^-1^·d^-1^, i.p. 15 min prior to CT on days 1, 3, 5 and 7	2.5 and 5 mg·kg^-1^·d^-1^ CBD attenuated the development of CT-induced mechanical allodynia.
Toth et al., (2010) [174]	CD1 mice	STZ Diabetes	0.1, 1 or 2 mg·kg^-1^·d^-1^, i.n. 3 months	1 and 2 mg·kg^-1^·d^-1^ CBD sig. attenuated the development of thermal and tactile hypersensitivity compared to 0.1 mg·kg^-1^·d^-1^ CBD.
			2 mg·kg^-1^·d^-1^, i.n. 1 month	CBD did not alleviate developed thermal or tactile hypersensitivity.
			1, 10 or 20 mg·kg^-1^·d^-1^, i.p. 3 months	20 mg·kg^-1^·d^-1^ CBD sig. attenuated the development of thermal and tactile hypersensitivity compared to 1 mg·kg^-1^·d^-1^ CBD.
			20 mg·kg^-1^·d^-1^, i.p. 1 month	CBD did not alleviate developed thermal or tactile hypersensitivity.
Costa et al., (2007) [41]	Wistar rats	CCI	2.5, 5, 10 or 20 mg·kg^-1^·d^-1^, oral 7 d	5, 10 and 20 mg·kg^-1^·d^-1^ CBD sig. decreased thermal and mechanical hyperalgesia on Tx day 7.
**Nociceptive (Inflammatory) Pain**				
Genaro et al., (2017) [70]	Wistar rats	Incision	0.3, 1, 3, 10 or 30 mg·kg^-1^, i.p.	3 mg·kg^-1^ CBD sig. decreased mechanical allodynia between 30- and 150-min post-Tx; 10 mg·kg^-1^ CBD sig. decreased mechanical allodynia 60 min post-Tx, only.
Hammell et al., (2016) [75]	Sprague-Dawley rats	FCA	0.6, 3.1, 6.2 or 62.3 mg·kg^-1^·d^-1^, t.c. 4 d	6.2 and 62.3 mg·kg^-1^ CBD sig. decreased pain-related behaviour on Tx day 4 and thermal hyperalgesia on Tx days 2, 3 and 4.
Costa et al., (2007) [41]	Wistar rats	FCA	20 mg·kg^-1^·d^-1^, oral 7 d	CBD sig. decreased thermal and mechanical hyperalgesia on Tx day 7.
Costa et al., (2004) [39]	Wistar rats	Carrageenan	5, 7.5, 10, 20 and 40 mg·kg^-1^, oral	5, 7.5, 10, 20 and 40 mg·kg^-1^·d^-1^ CBD sig. decreased thermal hyperalgesia 1–5 h post-Tx.
Costa et al., (2004) [40]	Wistar rats	Carrageenan	10 mg·kg^-1^, oral	CBD sig. decreased thermal hyperalgesia 1 h post-Tx.

The ‘Treatment Effects’ described are in comparison to a vehicle condition, unless otherwise stated

*CBD* Cannabidiol, *CCI* Chronic Constriction Injury, *CT* Chemotherapy, *FCA* Freund’s Complete Adjuvant, *i.n.* Intranasal, *i.t.* Intrathecal, *s.c.* Subcutaneous, *SNI* Spared Nerve Injury, *STZ* Streptozotocin, *t.c.* Transcutaneously, *Tx* Treatment

It is important to recognise that the analgesic effect of CBD likely depends on several factors, including the treatment dose and the type of pain involved. Indeed, low doses of CBD (e.g. ≤ 1 mg·kg^−1^, i.p.) do not consistently attenuate pain [29, 41, 70, 75, 101]; while higher doses are sometimes found to be more [29], and other times, less [70], efficacious than moderate doses in preclinical studies (Table 3). This highlights the importance of determining a therapeutic dose for CBD in analgesia. Data from King et al. [101] also demonstrate the selectivity of the response, indicating that CBD only effective in attenuating the development of neuropathic pain induced by certain chemotherapeutic agents (i.e. paclitaxel and oxaliplatin but not vincristine). Thus, placebo-controlled trials of CBD in treating pain in clinical populations and athletes are warranted.

**Table 3:** Clinical trials investigating the effect of CBD on subjective anxiety

Citation	Study design	Participants	Treatment(s)	Context	Treatment effect
**Healthy participants**					
Zuardi et al. (1993) [207]	DB (PC); BSD	I: 10; 23 y C: 10; 23 y	Oral 300 mg	Public speaking (90 min post-tx)	CBD sig. reduced SA post-public speaking. SA prior to, in anticipation of, and during speaking were unaffected.
Crippa et al. (2004) [45]	DB (PC); BSD	I: 5 M; 30 ± 5 y C: 5 M; 30 ± 5 y	Oral 400 mg	Low stress	CBD sig. reduced SA 60 and 75 min post-tx.
Bhattacharyya et al. (2010) [18]	DB (PC); WSD	15 M; 27 ± 6 y	Oral 600 mg	Low stress	SA was unaffected 1, 2 and 3 h post-tx.
Martin-Santos et al. (2012) [123]	DB (PC); WSD	16 M; 26 ± 5 y	Oral 600 mg	Low stress	SA was unaffected 1, 2 and 3 h post-tx.
Hindocha et al. (2015) [86]	DB (PC); WSD	48; 22 y	Vaporised 16 mg	Low stress	SA was unaffected 2, 30, 60. 90 and 120 min post-tx.
Zuardi et al. (2017) [208]	DB (PC); BSD	I: 12 (6 M); 23 ± 3 y I: 11 (5 M); 23 ± 3 y I: 12 (6 M); 23 ± 3 y C: 12 (6 M); 22 ± 2 y	I: Oral 100 mg I: Oral 300 mg I: Oral 900 mg	Public speaking (150 min post-tx)	300 mg CBD sig. reduced SA compared to 900 mg CBD (but not placebo) during public speaking. SA prior to, in anticipation of, and post-speaking were not affected by any treatment.
Linares et al. (2019) [116]	DB (PC); BSD	I: 15 M; 24 ± 3 y I: 15 M; 25 ± 3 y I: 12 M; 23 ± 3 y C: 15 M; 25 ± 4 y	I: Oral 150 mg I: Oral 300 mg I: Oral 600 mg	Public speaking (90 min post-tx)	300 mg CBD (but not 150 or 600 mg) sig. reduced SA during public speaking. SA prior to, in anticipation of, and post-speaking were not affected by any treatment.
**Clinical populations**					
Crippa et al. (2011) [44]	DB (PC); WSD SAD Participants	10 M; 24 ± 4 y	Oral 400 mg	Low stress	CBD sig. reduced SA 60, 75 and 140 min post-Tx.
Bergamaschi et al. (2011) [15]	DB (PC); BSD SAD Participants	I: 12 (6 M); 23 ± 2 y C: 12 (6 M); 23 ± 2 y	Oral 600 mg	Public speaking (90 min Post-Tx)	CBD sig. reduced SA during public speaking. SA prior to, in anticipation of, and post-speaking were not affected.
Hundal et al. (2017) [90]	DB (PC); BSD High Trait Paranoia	I: 16 (8 M); 26 ± 9 y C: 18 (8 M); 25 ± 8 y	Oral 600 mg	Virtual reality (130 min Post-Tx)	SA was unaffected.
Masataka (2019) [124]	DB (PC); BSD SAD Participants	I: 17 (8 M); 18–19 y C: 20 (8 M); 18–19 y	Oral 300 mg·d^-1^ (28-days)	Low stress	CBD sig. reduced SA 4-weeks post-tx.

*BSD* between-subject design, *C* control group, *CBD* cannabidiol, *DB* double-blind, *I* intervention group, *M* male subjects, *PC* placebo-controlled, *SAD* social anxiety disorder, *SA* subjective anxiety, *SB* single-blind, *Tx* treatment, *WSD* within-subject design

The “Treatment effects” described are in comparison to a placebo condition, unless otherwise stated

### Exercise-Induced Gastrointestinal (GI) Damage

While strenuous exercise increases blood supply to the active skeletal muscles, cardiopulmonary system and skin—other organs and tissues, including the GI tract, experience reduced oxygen and nutrient delivery [180]. If exercise is prolonged (e.g. >40 min), this “GI ischemia”, as well as the inflammation and oxidative stress that accompanies reperfusion, can compromise epithelial integrity [180]. Such effects may negatively influence exercise performance and post-exercise recovery due to GI distress (e.g. nausea, vomiting, abdominal angina, bloody diarrhoea) and impaired nutritional uptake [180].

CBD has demonstrated some effects that may be relevant to the management of exercise-induced GI damage. For instance, preclinical studies have shown that CBD (e.g. 0.01–10 mg·kg^−1^, i.v. or 10 mg·kg^−1^, i.p.) can attenuate tissue damage (e.g. reduce necrosis, blood concentrations of tissue damage markers and inflammation) induced by acute, peripheral ischemia-reperfusion (e.g. kidney, myocardium, liver) [55, 60, 62, 63, 130, 185] and colitis [23, 50, 154] in vivo; benefits that have generally been attributed to its reported antioxidant and anti-inflammatory effects (see also section “Exercise-Induced Muscle Damage—Muscle Function, Soreness, and Injury”) [50, 55, 60, 62, 63, 130, 154, 185]. Also, of interest is that CBD (1–100 μM) has been reported to restore intestinal permeability in vitro following exposure to *Clostridium difficile toxin A,* ethylenediaminetetraacetic acid and pro-inflammatory stimuli (e.g. interferon-gamma, TNF-α) [2, 3, 71].

Of course, it is important to recognise that evidence to support a therapeutic effect of CBD on GI damage in humans is currently lacking. In fact, two placebo-controlled, double-blinded clinical trials, one investigating the effect of CBD (10 mg·d^−1^; 56 days) on symptom severity in Crohn’s disease (*n* = 20) [133] and the other examining the impact of a “CBD-rich botanical extract” (250 mg·day^−1^ [4.7% THC]; 56 d) on the likelihood of remission in ulcerative colitis (*n* = 60) [94], have so far been unable to demonstrate a protective effect of CBD (above placebo) on disease markers, including C-reactive protein, faecal calprotectin, and pro-inflammatory cytokines (e.g. IL-2, IL-6, TNF-α).

While CBD could potentially attenuate exercise-induced GI damage, it is important to note that other anti-inflammatory agents, such as the NSAID, ibuprofen, have been reported to exacerbate exercise-induced GI damage and impair gut barrier function [181]. The precise mechanism(s) underpinning these effects have not been fully elucidated. However, NSAIDs have been suggested to augment GI ischemia by inhibiting the COX1 and COX2 enzymes and interfering with nitric oxide production [180]. Some in vitro research similarly suggests that CBD partially inhibits COX1 and COX2, although this effect has only been reported at supraphysiological concentrations (e.g. 50–500 μM CBD) [92]. Thus, the effect of CBD on exercise-induced GI damage warrants clarification.

### Bone Health

While the beneficial effects of high-impact exercise on bone health are well established [38], other factors within the sporting context (e.g. traumatic injuries, low energy availability [117]) may cause or contribute to reduced bone health and the development of fractures in athletes.

A small number of preclinical studies have investigated the effects of CBD on bone structure and function [103, 112, 135]. While most have used animal models that are limited in their direct relevance to sport and/or exercise performance (e.g. periodontitis, systemic skeletal degeneration due to spinal cord injury) one investigation [103] did report that CBD improved the healing of femoral fractures in Sprague-Dawley rats. Specifically, chronic CBD treatment (i.e. 5 mg·kg^–1^·day^–1^, i.p.) decreased callus size 4-weeks post-fracture and enhanced the biomechanical properties of the bone at 8-weeks (i.e. maximal force, work-to-failure on a 4-point bending test) [103]. While the mechanism(s) underlying this effect require clarification, CBD may act to inhibit expression of RANK and RANK-L (i.e. indicative of an effect to suppress osteoclastogenesis, and thus, bone resorption) and decrease the production of pro-inflammatory cytokines (e.g. IL-1β, TNF-α) at the site of injury [103, 135]. Evidence that activation of CB_2_R induces bone matrix deposition has also recently emerged [150] alongside data suggesting that CBD may have partial agonist effects at this site [171]. These initial findings suggest that further research investigating the effect of CBD on acute skeletal injuries is worthwhile.

### Cardiovascular (CV) and Metabolic Functions

A number of studies have measured CV responses to CBD (100–1200 mg) in humans and, overall, it appears that resting HR is unaffected (see Sultan et al. [165] for review). However, some evidence does suggest that CBD (600 mg) reduces resting systolic BP (e.g. –6 mmHg) [95, 164]. Preclinical studies have likewise shown that CBD influences vascular function [139, 161, 162, 193, 194]. Briefly, in vitro CBD treatment (i.e. ≤ 2 h exposure to 1–10 μM) has been reported to induce vasorelaxation [139] and potentiate vasorelaxation to acetylcholine [162, 193] in isolated (pre-constricted) arteries of rats [139, 162, 193]. A recent study [161] also found that in vitro CBD treatment (i.e. ≤ 2 h exposure to 10 μM) induced ~40% vasorelaxation in isolated (pre-constricted) mesenteric arteries of humans with various clinical conditions (e.g. cancer, inflammatory bowel disease, type Z diabetes mellitus [T2DM]).

In addition to “resting” CV parameters, a recent meta-analysis (of largely preclinical studies) found that CBD attenuated “stress-induced” (e.g. via fear-conditioning or physical-restraint) increases in HR and BP (BP −3.5 mmHg; 95% CI −5.2, −1.9; *I*^2^ = 73%; HR −16 mmHg; 95% CI −26, −6; *I*^2^ = 92%) [165], that said, most studies measuring CV responses to CBD (150–600 mg) under “stress-inducing” conditions in humans (e.g. public speaking) find no effect on HR or BP [15, 116, 207]. One placebo-controlled, double-blinded (single-dose) crossover trial of healthy males (*n* = 9) [95] did report that CBD (600 mg) increased HR in the presence of certain stressors (i.e. a mental arithmetic test, an isometric contraction on a hand-grip dynamometer, and cold exposure); and, at times, reduced systolic and diastolic BP. However, these differences were apparent at baseline (pre-stress) and the data were not standardised to account for this, making interpretation difficult.

Taken together, these findings suggest that CBD has the potential to influence CV function. However, the implications of these effects in regard to exercise performance are unclear. Studies investigating the effect of CBD on exercise-induced CV responses are therefore required to clarify its utility within the sport and exercise context.

One final observation to note is that some initial data suggest CBD might influence mitochondrial function. Indeed, in vivo CBD treatment has been reported to increase the activity of mitochondrial complexes [48, 77, 130, 178] (30–60 mg·kg−^1^, i.p. acute or chronic 14 days Wistar rats; 3 and 10 mg·kg^−1^, i.v. C57BL/6 J mice; 10 mg·kg^−1^·day^−1^, i.p. 5 days C57BL/6 J mice; and 10 mg·kg^−1^·day^−1^, i.p. 14 days Wistar rats) in various tissues and models (i.e. healthy brain, myocardium following doxorubicin treatment, brain following neonatal iron-overload, hepatic ischemia-reperfusion injury) and increase mitochondrial biogenesis [77] (10 mg·kg^−1^·day^−1^, i.p. 5 days C57BL/6 J mice; myocardium following doxorubicin treatment). Such effects could have implications for energy metabolism during exercise.

### Thermoregulation

Heat loss mechanisms play a pivotal role in the maintenance of homeostasis during exercise; any treatment or condition that alters core body temperature (T_C_), therefore has the potential to impact exercise performance [192]. The effect of CBD on T_C_ has been investigated in rodents [65, 82, 83, 85, 96, 118, 166, 182]. The most recently published study found that CBD (100 mg·mL^−1^, 30 min vaporised; Wistar rats) reduced T_C_ (−1.0 °C) 60 and 90 min following inhalation in resting animals [96]. In contrast, Long et al. [118] reported a hyperthermic effect (+2.0 °C) 30 min post-treatment (1 and 10 mg·kg^−1^, i.p.; C57BL/6JArc mice) during a chronic-dosing experiment, although, this response was only observed intermittently during a 21-day protocol (e.g. ~8% of total measurements). The fact that CBD affected T_C_ in these experiments is difficult to explain, since although other cannabinoids (e.g. Δ^9^-THC, AEA) have demonstrated a capacity to moderate T_C_ when administered exogenously (e.g. low doses of Δ^9^-THC may sometimes induce hyperthermia [168] and high doses cause hypothermia [65, 82, 83, 85, 96, 118, 166, 182]), these effects occur via a CB_1_R-mediated mechanism [42, 166, 196]. In addition to this, no other studies appear to have detected changes in T_C_ with CBD administration [65, 82, 83, 85, 166, 182].

Overall, despite some inconsistencies, the available data suggest that CBD is unlikely to have a major influence on T_C_ or thermoregulatory processes. In any case, it seems that with the exception of self-reported feelings of “coldness” [33, 88], exogenous cannabinoids do not typically induce the same overt, significant effects on T_C_ in humans [104, 183] as are seen in rodents. Still, it should be acknowledged that the thermoregulatory response to heat stress (i.e. passive or metabolic), specifically, has not been studied.

### Dietary Intake and Feeding

An adequate intake of energy and nutrients is essential to support optimal athletic training, recovery, and performance [173]. Various preclinical studies have investigated the effect of CBD on feeding behaviour in rodents [58, 93, 155, 159, 195], with results suggesting that higher doses may influence food intake several hours post-treatment. Indeed, while CBD, at doses of 3–100 mg·kg^−1^, i.p. (IRC mice) [195] and 1–20 mg·kg^−1^, i.p. (Wistar rats) [155], failed to influence food intake during a 1 h ad libitum feeding period, moderate to high doses of CBD (4.4 mg·kg^−1^, i.p. [58]. and 50 mg·kg^−1^, i.p. [159]) suppressed food intake (in rats) during longer ad libitum feeding periods (i.e. 4–6 h). In line with these results, Ignatowska-Jankowska et al. [93] found that chronic CBD treatment (2.5 and 5 mg·kg^−1^·day^−1^, i.p. 14 days) attenuated BM gains in growing Wistar rats. A recent systematic review of human trials also reported that individuals with epilepsy receiving CBD (5–20 mg·kg^−1^·day^−1^) were more likely to experience decreased appetite than those receiving placebo (i.e. ~20 vs. 5% of patients) [107].

That said, a mechanistic understanding of these effects of CBD on feeding behaviour remains to be established. Other cannabinoids with CB_1_R agonist effects (e.g. Δ^9^-THC, AEA, cannabinol) reliably induce hyperphagia when administered exogenously [58, 197, 198]; but CBD lacks such an effect. Ignatowska-Jankowska et al. [93] did report that the selective CB_2_R antagonist, AM630, prevented CBD-induced BM changes; however, CB_2_R has not generally been linked to feeding behaviour, and if CBD is indirectly increasing endocannabinoid tone (i.e. via AEA) [92], this might be expected to promote feeding behaviour (via indirect CB_1_R agonist effects) [197]. A role for GI side effects in affecting appetite therefore cannot be ruled out [107]. Further preclinical research appears to be required to clarify the mechanisms underlying these functional effects on feeding. Controlled trials are also needed to determine whether CBD influences appetite and dietary behaviour in humans, particularly during the pre- and post-exercise period, where nutrient provision is critical.

### Illness and Infection

Some research suggests that athletes experience a decrease in immunity and are at increased risk of developing acute illnesses (particularly upper respiratory tract infections) during periods of heavy training and competition [184]. This phenomenon has been attributed to various factors such as increased psychological stress, poor sleep, long-haul travel, exposure to extreme environments (e.g. altitude), and low energy availability [184]. A recent review of online content identified a number of webpages claiming benefits of using CBD for the treatment of viral illnesses, including cold and flu [167]. However, research supporting such “protective effects” of CBD is extremely limited. In fact, the authors identified only two (in vitro) studies reporting anti-microbial effects [5, 179] and two (in vitro) studies reporting anti-viral effects [119, 122] (see Tagne et al. [167] and Nichols et al. [136] for reviews). In the former, CBD demonstrated anti-microbial activity against various strains of *Staphylococcus aureus* [5, 179], as well as *Staphylococcus pyogenes*, *Staphylococcus milleri*, and *Staphylococcus faecalis* [179] at minimum concentrations of ~3.2–15.9 μM; however, one study also found that these effects were virtually abolished when the original media (a nutrient broth agar) was replaced with one containing 5% blood (increasing the minimum concentration to ~160 μM CBD) [179]. In the latter, CBD indicated anti-viral activity against the hepatitis C virus (EC_50_ = 3.2 μM) and the Kaposi’s sarcoma-associated herpesvirus (EC_50_ = 2.1 μM), but not the hepatitis B virus [119, 122]. While these findings hint at some promise, others caution that CBD could potentially weaken host defence against invading pathogens because of its tendency to modify the function of various immune cells (see also section “Exercise-Induced Muscle Damage—Muscle Function, Soreness, and Injury”) [136, 147]. Importantly, a systematic review of studies investigating the safety of CBD in individuals with intractable epilepsy found that upper respiratory tract infections were similarly infrequent in participants who received the active treatment (5–20 mg·kg^−1^·day^−1^) and placebo (approx. 10% of individuals) [107]. Further research to develop a better understanding of “if” and “how” CBD influences the development and progression of illness and infection in both athlete and non-athlete populations would be useful.

### Sports Performance Anxiety (SPA)

High levels of pre-competition stress, or sports performance anxiety (SPA) [141], can be detrimental to athletic performance [43]. This impairment has been attributed to both the direct (i.e. anxiogenic) and indirect (e.g. decreased nutritional intake, increased energy expenditure, loss of sleep) effects of SPA [28]. While behaviour therapies (e.g. cognitive behavioural therapy) are the preferred treatment, a combination of pharmaceutical and psychological interventions may be indicated in some cases [141].

A number of (small) clinical trials have investigated the effect of CBD on subjective anxiety in healthy individuals [18, 45, 86, 116, 123, 207, 208] and in individuals with social anxiety disorder (SAD) [15, 44, 124] and high trait paranoia [90] under both standard (i.e. “low stress”) [18, 44, 45, 86, 123, 124] and “stress-inducing” (e.g. simulated public speaking) [15, 90, 116, 207, 208] conditions (Table 3). Overall, results suggest that CBD has little influence on anxiety under “low stress” conditions in healthy participants [18, 86, 123]. However, several studies have demonstrated anxiolytic effects of CBD (300–600 mg) under “stress-inducing” conditions in both healthy participants and those with SAD [15, 44, 116, 124, 207]. In fact, CBD (300 mg) had comparable efficacy to the anxiolytic 5-HT_1A_ agonist drug, ipsapirone (5 mg), during a simulated public speaking test in one study [207]. On the other hand, some other clinical investigations (involving similar “stress-inducing” stimuli) have found no effect of CBD [90, 208] and Hundal et al. [90] observed a non-significant trend (*p* < 0.10) towards an anxiogenic effect with 600 mg CBD in a high trait paranoid group (*n* = 32). Inconsistencies could be due to inter-individual differences in baseline anxiety levels and the magnitude of the stress-response to the stressor imposed, small sample sizes, and differences in the dose and formulation of CBD provided. Indeed, Linares et al. [116] observed an inverted U-shaped dose-response relationship between acute CBD treatment and subjective anxiety, indicating that 300 mg (Hedges’ *g* = 1.0) had a stronger anxiolytic effect than 150 mg (Hedges’ *g* = 0.7) or 600 mg (Hedges’ *g* = 0.6).

Taken together, it appears that moderate doses of CBD may be anxiolytic in stressful situations and in individuals with SAD. Thus, studies investigating the effect of CBD (in conjunction with behaviour therapies) on pre-competition anxiety, as well as nutritional intake, energy expenditure, symptom perception during exercise (e.g. ratings of perceived exertion), and sleep in athletes who are negatively impacted by SPA are warranted.

### Sleep

The importance of adequate sleep in facilitating optimal athletic performance and recovery is increasingly recognised [121]. Yet, athletes often sleep less (e.g. ~6.5–6.7 h·night^−1^) and experience poorer quality sleep than non-athletes [79, 109, 152]. Factors that contribute to poor sleep among athletes include evening competitions and training sessions, pre-competition anxiety, use of caffeine, and long-haul travel (e.g. jet lag, travel fatigue) [121].

Several studies have investigated the effect of CBD on sleep in humans [26, 32, 115, 157]. The first placebo-controlled, double-blinded (single-dose) crossover trial [26] found that 160 mg CBD (but not 40 or 80 mg) increased self-reported sleep duration in individuals with insomnia (*n* = 15); although time to sleep onset, number of sleep interruptions, and likelihood of experiencing “good sleep” were unchanged. Two case studies also indicated the benefits of CBD [32, 157]. Specifically, Chagas et al. [32] observed a reduction in symptoms of rapid eye movement sleep-behaviour disorder in four individuals with Parkinson’s disease (75–300 mg·day^−1^; 42 day) and Shannon et al. [157] found that CBD (~25 mg·day^−1^) improved subjective sleep quality in a young girl with post-traumatic stress disorder (PTSD). Of course, these studies [26, 32, 157] are limited in that they rely on subjective measures of sleep and involve small sample sizes; the improvements observed could also be due to CBD attenuating other sleep-impairing comorbid conditions (e.g. anxiety, PTSD). Indeed, a recent placebo-controlled, double-blinded (single-dose) crossover trial [115] found no effect of CBD (300 mg) on sleep architecture measured via polysomnography in healthy adults (*n* = 27).

While CBD seems unlikely to directly influence sleep in healthy humans [115] (and may be “sleep-promoting” in those with certain comorbid conditions) [26, 32, 157], a small number of rodent studies suggest that the cannabinoid could actually be “wake-inducing” [128, 132, 204]. One placebo-controlled, double-blinded (single-dose) crossover trial of healthy individuals (*n* = 8) [137] also found that low-dose CBD (15 mg) counteracted some of the sedative effects of co-administered Δ^9^-THC (15 mg), i.e. increasing overnight wakefulness; although, this effect could be due to CBD acting as a NAM of CB_1_R, thereby attenuating Δ^9^-THCs effects on that receptor [92]. Differences in the doses of CBD administered might partly explain this inconsistency. Indeed, Monti [128] observed a biphasic effect of CBD on sleep in Wistar rats, such that a lower dose decreased, and a higher dose increased (20 vs. 40 mg·kg^−1^, i.p.), slow-wave sleep latency. However, inter-species differences are also a consideration as nocturnal animals appear to exhibit different circadian patterns of endocannabinoid signaling compared to humans [84, 131]. Collectively, the current evidence on CBD and sleep endorses the need for further research in clinical populations and athletes.

### Cognitive and Psychomotor Function

A small number of clinical trials have investigated the effects of CBD on cognitive and psychomotor function in healthy individuals (Table 4). Overall, results suggest a minimal influence of CBD on cognitive or psychomotor function. While one early investigation [98] reported that CBD (15 or 60 mg) caused participants to under-/over-estimate the duration of a 60-s interval on several occasions throughout a repetitive testing protocol (i.e. as is indicative of impaired concentration), the magnitude of error was small (e.g. <5 s; particularly, in comparison to that observed with Δ^9^-THC, e.g. ~10–25 s [98]) and the effect was inconsistent, suggesting it may be an artefact of the between-subject design and small sample size. A more recent investigation [86] observed an improvement in emotion recognition with CBD (16 mg vaporised); however, this “ability” may have limited relevance to the sporting context. More applicable, perhaps, is Dalton et al. [49] finding no effect of CBD (150 μg·kg^−1^ vaporised) on balance or coordination (a product of both cognitive and motor function). A more recent investigation likewise found no effect of oral or vaporised CBD (100 mg) on cognitive performance between 30 min and 8 h post-treatment [160] (Table 4). Thus, while only a narrow range of doses and relatively few discrete cognitive functions have been studied, the available data suggest that CBD is unlikely to impact cognitive or psychomotor function on healthy individuals.

**Table 4:** Clinical trials investigating the effect of CBD on cognitive and psychomotor function in healthy individuals

Citation	Study design	Participants	Treatment(s)	Cognitive tasks	Treatment effect
Karniol et al. (1974) [98]	DB (PC); BSD	I: 5 M; 21–34 y I: 5 M; 21–34 y I: 5 M; 21–34 y C: 5 M; 21–34 y	I: Oral 15 mg I: Oral 30 mg I: Oral 60 mg	Time estimation task^a^	15 mg CBD sig. altered the ETI (without FB) at 45 and 95 min. 60 mg CBD sig. altered the ETI (without FB) at 95 and 180 min. No other sig. differences were observed.
Dalton et al. (1975) [49]	DB; WSD	15 M; 21–24 y	Vaporised 150 μg·kg^-1^	Wobble board Pursuit meter Delayed auditory feedback “Pegs”	Standing steadiness, hand-eye coordination, cognition and manual coordination were unchanged from baseline 5–85 min post-tx
Leweke et al. (2000) [111]	SB; WSD	9 M; 26–35 y	Oral 200 mg	Binocular depth inversion	Depth inversion scores were unchanged from baseline 1, 2, 3, 4, 5, 6 and 24 h post-tx.
Bogwardt et al. (2008) [22]	DB (PC); WSD	15 M; 27 ± 6 y	Oral 600 mg	Go/No-Go Task	Reaction time and accuracy were unaffected between ~1 and 2 h post-tx.
Bhattacharyya et al. (2009) [17]	DB (PC); WSD	15 M; 27 ± 6 y	Oral 600 mg	Verbal memory task	Word recall was unaffected ~1–2 h post-tx.
Palo Fusar-Poli et al. (2009) [67]	DB (PC); WSD	15 M; 27 ± 6 y	Oral 600 mg	Facial emotion recognition	Reaction time and accuracy were unaffected ~1–2 h post-tx.
Hindocha et al. (2015) [86]	DB (PC); WSD	48 (34 M); ~22 y	Vaporised 16 mg	Facial emotion recognition	CBD sig. improved emotion recognition at 60% intensity 10 min post-tx. Recognition at 20, 40, 80 and 100% intensity were unaffected.
Hundal et al. (2017) [90]	DB (PC); BSD	I: 16 (8 M); 26 ± 9 y C: 18 (8 M); 25 ± 8 y	Oral 600 mg	Symbol coding Digit span (forward and reverse) Verbal learning task	Digit-symbol recoding, forward digit span, reverse digit span, immediate recall and delayed recall were unaffected 135 min post-tx.
Arndt & de Wit (2017) [8]	DB (PC); WSD	38 (19 M); 24 ± 4 y	Oral 300, 600 and 900 mg	Emotional stroop Facial emotion recognition Attentional bias task	Reaction time, emotion recognition and attentional bias were unaffected 3–4 h post-tx.
Birnbaum et al. (In Press) [19]	No blind; WSD	8 (6 M); 49 y	Oral 300 mg	Phonemic and semantic fluency Digit span Trail making test (A & B) Symbol–digit modality task	Test scores were unchanged from baseline 2.5 h post-tx (test outcomes not specified)
Spindle et al. (In Press) [160]	DB (PC); WSD	18 (9 M); 31 ± 6 y	Oral 100 mg and Vaporised 100 mg	Digit symbol substitution Divided attention task Paced serial addition task	Speed and accuracy of digit symbol substitution and paced serial addition, as well as speed, accuracy and tracking on the divided attention task were unaffected between 0.5 and 8 h post-tx.

*BSD* between-subject design, *C* control group, *CBD* cannabidiol, *DB* double-blind, *ETI* estimated time interval, *FB* feedback, *I* intervention group, *M* male subjects, *PC* placebo-controlled, *SB* single-blind, *Tx* treatment, *WSD* within-subject design

The “Treatment effects” described are in comparison to a placebo condition, unless otherwise stated

^a^Time estimation task: participants were asked to produce a 60 s interval 10 times. The first 5 estimations were without feedback and the remaining 5 were with feedback. This task was repeated 45, 95. and 180 min post-treatment

## Other Considerations

### CBD “Nutraceutical” Products

Over-the-counter CBD-containing “nutraceuticals” are now readily available in many countries and of increasing interest to the community [108]. However, it is important to be aware that these products are often limited in that they typically contain low levels of CBD (e.g. ~10-50 mg·mL^−1^), [20] making it difficult to achieve the higher doses often used in the studies described in this review.

Additionally, over-the-counter CBD nutraceuticals are not always manufactured to the same pharmaceutical standards as regulated, prescription CBD products (e.g. Epidiolex®). Indeed, a recent study [20] reported that only ~31% of CBD extracts sold online (*n* = 84) were “labelled accurately” (i.e. a measured CBD content within 10% of the labelled value); nearly half underestimated, and a quarter overestimated, their CBD content—approximately one-fifth of samples also contained detectable levels of Δ^9^-THC. A similar investigation [148] of nine CBD “e-liquids” (used for vaporising) found that two contained Δ^9^-THC, four contained 5-fluoro MDMB-PINACA (a potent synthetic cannabinoid receptor agonist with powerful psychoactive effects), and one contained dextromethorphan. Thus, individuals using these products are at risk of over-/under-dosing, adverse health effects and/or possibly, recording a positive drug test result. This suggests that until such time as better manufacturing standards are imposed, athletes in competition might wish to avoid using non-regulated CBD-containing nutraceuticals, or, at least carefully investigating their quality control and provenance before using them.

### Conversion of CBD to Δ^9^-THC

In vitro *studies* have shown that CBD can undergo conversion to Δ^9^-THC with prolonged exposure to simulated gastric fluid [126, 189]. However, this effect has not been observed in vivo. When investigating the phenomenon in minipigs (30 mg CBD·kg^−1^·day^−1^, oral 5 days), which, like humans, have omnivorous diets and other GI similarities (e.g. pH, transit time, drug absorption), Wray et al. [202] found no detectable (i.e. <0.5 ng·mL^−1^) Δ^9^-THC or 11-OH-THC in any plasma or GI tract samples collected. Consroe et al. [37] also found no detectable Δ^9^-THC (i.e. <0.5 ng·mL^−1^) in human plasma following chronic high-dose CBD treatment (700 mg·day^−1^; 30 days). These data suggest that pure CBD is unlikely to produce a positive drug test result (i.e. indicated by a urinary THC–COOH level > 150 ng·mL^−1^ [200]) or any intoxicating (ergolytic) effects due to conversion to Δ^9^-THC. To date, however, no studies have directly investigated whether CBD can elicit a positive drug test result in athletes.

## Conclusions

CBD has been reported to exert a number of physiological, biochemical, and psychological effects, that have the potential to benefit athletes. For instance, there is preliminary supportive evidence for anti-inflammatory, neuroprotective, analgesic, and anxiolytic actions of CBD and the possibility it may protect against GI damage associated with inflammation and promote the healing of traumatic skeletal injuries. However, it is important to recognise that these findings are very preliminary, at times inconsistent, and largely derived from preclinical studies. Such studies are limited in their generalisability to athletes (and humans in general), and often administer high doses of CBD that may be difficult to replicate in humans. The central observation is that studies directly investigating CBD and sports performance are lacking, and until these are conducted, we can only speculate in regard to its effects. Nonetheless, this review suggests that rigorous, controlled investigations clarifying the utility of CBD in the sporting context are clearly warranted.

## Data Availability

Not applicable.

## Abbreviations

Δ^9^-THC: Δ^9^-tetrahydrocannabinol
5-HT_1A_: Serotonin 1A receptor
AEA: Anandamide
CV: Cardiovascular
CBD: Cannabidiol
CB_1_R: Cannabinoid CB_1_ receptor
CB_2_R: Cannabinoid CB_2_ receptor
*C*_max_: Maximum plasma concentrations
COX: Cyclooxygenase
CYP: Cytochrome P
EIMD: Exercise-induced muscle damage
HI: Hypoxic ischemia
FAAH: Fatty acid amide hydrolase
GI: Gastrointestinal
GPR: G protein-coupled receptor
IL: Interleukin
i.p.: Intraperitoneal
i.v.: Intravenous
NAM: Negative allosteric modulator
NSAID: Non-steroidal anti-inflammatory drug
p.o.: Oral
SPA: Sports performance anxiety
SAD: Social anxiety disorder
TBI: Traumatic brain injury
T_C_: Core body temperature
*t*_max_: Time to reach maximum plasma concentrations
TRVP_1_: Transient potential vanilloid receptor type 1 channel
TNF: Tumour necrosis factor
USA: United States of America

## Footnotes

1 E.g. 10–30 mg·kg^−1^·day^−1^, i.p. 3 days [129, 153], 1 mg·kg^−1^·day^−1^, i.v. 3 days [13], 1–3 mg·kg^−1^, i.p. pre- and 3 h post-HI [81, 83] or 0.1 mg·kg^−1^, i.v. 15 and 240 min post-HI [4].
